# Fatigue is associated with excess mortality in the general population: results from the EPIC-Norfolk study

**DOI:** 10.1186/s12916-016-0662-y

**Published:** 2016-08-20

**Authors:** Neil Basu, Xingzi Yang, Robert N. Luben, Daniel Whibley, Gary J. Macfarlane, Nicholas J. Wareham, Kay-Tee Khaw, Phyo Kyaw Myint

**Affiliations:** 1 Epidemiology Group, Institute of Applied Health Sciences, School of Medicine & Dentistry, University of Aberdeen, Foresterhill, Aberdeen, Scotland AB25 2ZD UK; 2 Department of Public Health and Primary Care, Strangeway Research Laboratory, University of Cambridge, Cambridge, UK; 3 Department of Public Health and Primary Care, Clinical Gerontology Unit, University of Cambridge, Cambridge, UK; 4 MRC Epidemiology Unit, Cambridge, UK

**Keywords:** Fatigue, Mortality, Cardiovascular, Cancer

## Abstract

**Background:**

Significant fatigue is a frequent reason for seeking medical advice in the general population. Patients, however, commonly feel their complaint is ignored. This situation may be because clinicians perceive fatigue to be benign, unrelated to traditional biomedical outcomes such as premature mortality. The present study aimed to investigate whether an association between significant fatigue and mortality actually exists, and, if so, to identify potential mechanisms of this association.

**Methods:**

A population-based cohort of 18,101 men and women aged 40–79 years who completed a measure of fatigue (Short Form 36 vitality domain, SF36-VT) in addition to providing information on possible confounding factors (age, sex, body mass index, marital status, smoking, education level, alcohol consumption, social class, depression, bodily pain, diabetes, use of β blockers, physical activity and diet) and mechanisms (haemoglobin, C-reactive protein and thyroid function) were followed up prospectively for up to 20 years. Mortality from all causes, cancer and cardiovascular disease was ascertained using death certification linkage with the UK Office of National Statistics.

**Results:**

During 300,322 person years of follow-up (mean 16.6 years), 4397 deaths occurred. After adjusting for confounders, the hazard ratio (HR) for all-cause mortality was 1.40 (95 % confidence interval [CI] 1.25–1.56) for those reporting the highest fatigue (bottom SF36-VT quartile) compared with those reporting the lowest fatigue (top SF36-VT quartile). This significant association was specifically observed for those deaths related to cardiovascular disease (HR 1.45, 95 % CI 1.18–1.78) but not cancer (HR 1.09, 95 % CI 0.90–1.32). Of the considered mechanisms, thyroid function was most notable for attenuating this association. The risk of all-cause mortality, however, remained significant even after considering all putative confounders and mechanisms (HR 1.26, 95 % CI 1.10–1.45).

**Conclusions:**

High levels of fatigue are associated with excess mortality in the general population. This commonly dismissed symptom demands greater evaluation and should not automatically be considered benign.

**Electronic supplementary material:**

The online version of this article (doi:10.1186/s12916-016-0662-y) contains supplementary material, which is available to authorized users.

## Background

Fatigue is a normal experience, but for some persons its impact is profound, resulting in concerns regarding their health. In fact, fatigue is one of the commonest complaints reported in primary care, constituting up to 20 % of all presentations [1, 2] and the principal reason for consultation in 5-7 % of cases [1–3].

The psychosocial consequences of fatigue are becoming increasingly recognised. It is strongly associated with poor long-term quality of life [4] and work ability [5]. Indeed, excessively fatigued workers are estimated to cost employers $136.4 billion/year in the USA alone [6]. Despite this, patients’ commonly consider the symptom to be ignored by medical practitioners [7] and approximately 75 % of these patients are not followed up [8]. It may be that doctors are justifiably selective in their follow-up of these patients; however, this paucity of attention may also reflect the perception that fatigue does not affect traditional biomedical outcomes such as premature mortality.

A few studies have reported a possible association between fatigue and mortality, although these suffer from significant methodological limitations: (1) small sample sizes which prohibit the inspection of cause-specific mortality, (2) non-generalisable samples, (3) the use of non-validated single item measures of fatigue which preclude evaluation of dose response and (4) a limited consideration of potential confounders and mechanisms, clearly crucial in the context of such a multifactorial symptom [9, 10].

So why could fatigue result in premature mortality? Some have plausibly hypothesised that fatigue leads to harmful changes in behaviour [9]. Alternatively, the underlying pathophysiology of the symptom itself could lead to poor outcomes – fatigue is a commonly recognised feature of dysfunctional physiology, for example it is a cardinal feature of low rates of metabolism due to hypothyroidism, which in turn is an established risk factor of cardiovascular disease (CVD)-related mortality [11].

The aim of this study was to describe and then understand the reasons for an association between fatigue and mortality (both all-cause and cause-specific) in a large prospective population-based cohort.

## Methods

### Study design and sample

Participants were drawn from the European Prospective Investigation into Cancer and Nutrition (EPIC)-Norfolk study. The study methodology and sample distribution have been previously reported in detail [12]. In brief, this prospective cohort study recruited members of the general population, aged 40–79 years at the baseline, using general practice age-sex registers from the region of Norfolk – a relatively stable and immobile population in the UK. Participants attended for baseline health assessments between 1993 and 1997 and have since been followed up by way of additional health assessments, questionnaires and national registry data linkage. Ethical approval was obtained from the Norwich Research Ethics Committee.

### Prognostic factor of interest

Investigating the determinants of mortality is one of the aims of this cohort. In this specific study, the prognostic factor of interest was fatigue, defined according to the UK version of the Short Form 36 vitality domain (SF36-VT). Although there is no single accepted measure of fatigue, the SF36-VT is commonly used and validated in studies of both diseased and general populations [13, 14]. In fact, it is employed as a gold standard tool to support the development of other fatigue assessment instruments [15]. Collected within the 18-month follow-up study questionnaire, the SF36-VT consists of four questions which refer to the previous 4 weeks: (1) Did you feel full of life? (2) Did you have a lot of energy? (3) Did you feel worn out? (4) Did you feel tired? These were summed and transformed into a 0–100 scale (where a high score represented low fatigue levels) using established scoring algorithms [14]. For the purposes of analysis, the final scores were categorised according to quartiles derived from the whole sample.

### Outcome ascertainment

Mortality was ascertained using death certification linkage with the UK Office of National Statistics (ONS) at follow-up (censored in 2013). In addition to data on all-cause mortality, deaths specifically attributed to CVD (defined by International Classification of Disease [ICD], 9:401–448 or ICD 10:I10-I79) or cancer (defined by ICD 9:140–208 or ICD 10:C00-C97) were recorded.

### Putative confounders/mechanisms

Multiple putative covariates, commonly considered by clinicians when evaluating the complaint of fatigue, were measured at baseline.

The mechanisms of fatigue are poorly described and some may also be considered confounders. We undertook a conservative approach by assuming that a factor fulfilling the statistical criteria for a confounding variable (i.e. a variable that is significantly associated with both fatigue and mortality) was a confounder. A covariate not fulfilling such criteria was considered to be a possible mechanism.

The covariates were collected by trained staff according to standardised protocols:

#### Self-reported measures

A survey capturing demographic and lifestyle information was conducted at baseline. This included age, gender and marital status. As with previous EPIC analyses, social class was categorised according to the Registrar General’s occupation classification scheme and further collapsed into manual or non-manual categories [16]. Education status of participants was defined according to whether or not they reached A-level standard (the senior secondary education examination in England). Self-reported total alcohol consumption was quantified as the total units of alcohol (1 unit equates about 8 g) consumed in a week and smoking as current/non-current. In order to ascertain the prevalence of baseline morbidity, participants were asked ‘Has your doctor ever told you have the following?’ followed by a list of illnesses which included cancer, diabetes, depression, heart attack and stroke. The SF36 bodily pain domain (simultaneously collected with the SF36-VT) was scored and transformed to a 0–100 scale employing validated methods [14]. As with SF36-VT, a high score represented a good health state, that is, low pain. Physical activity was assessed using the EPIC-validated questionnaire [17] which rated participants as inactive, moderately inactive, moderately active or active. The consumption of fruit and vegetables (g/day) was determined by the EPIC Food Frequency Questionnaire, also a previously well-described and validated tool [18] which asks participants to quantify their average consumption of a number of food items over the past year. Medication use, including aspirin, was self-reported and captured in the baseline survey.

#### Clinical measures

Height and weight were assessed and the body mass index subsequently calculated (weight[kg]/(height[m^2^]). This has been categorised according to World Health Organisation definitions [19].

Non-fasting blood samples were collected. AutoDELFIA time-resolved fluoroimmunoassay kits (Wallac, Finland) were used to assay thyroid-stimulating hormone (TSH) levels (hypothyroid status: >4.0 mU/l) in order to evaluate thyroid status; serum concentrations of the inflammatory marker C-reactive protein (CRP) were measured using a high-sensitivity assay on an Olympus AU640 Clinical Chemistry Analyser (Olympus UK Ltd, Watford, UK); and a Coulter MD18 Haematology Analyzer (Addenbrookes Hospital, Cambridge, UK) measured haemoglobin (Hb) status to enable assessment for anaemia (moderate anaemia defined as Hb < 110 g/L [20]).

### Statistical analysis

The cross-sectional relationship between quartiles of fatigue severity and other putative confounders was first described using Chi-squared tests (for categorical confounder data) and ANOVA (for continuous confounder data), as appropriate.

Cox proportional hazards models were then employed to examine the longitudinal relationships between quartiles of fatigue and subsequent all-cause mortality over the follow-up period. First, an unadjusted model was developed (model A). Then, participants with a history of significant CVD (stroke and/or myocardial infarction) or cancer at baseline were excluded in order to avoid major confounding (model B).

Using simple descriptive statistics, as appropriate, the associations between the covariates of interest and fatigue and mortality, respectively, were calculated. Those covariates statistically associated (*p* < 0.05) with both SF36 vitality and mortality were considered to be confounders and those not were considered as possible mechanisms. Mechanistic factors are on the causal pathway between an exposure and an outcome; if adjusted for in the analysis, they will attenuate the observed exposure–outcome association. We tested our proposed ‘putative mechanisms’ by adjusting for them in the analysis with the expectation that if they are indeed mechanisms then the magnitude of association between fatigue and mortality will be reduced.

Confounders were grouped and introduced (model C) and then the influence of each possible mechanism was evaluated by submitting these, in isolation, to this model. Subsequently, all putative confounders and mechanisms were advanced together in a single model.

To better determine the precise causes of any observed mortality, the modelling process was repeated to examine the longitudinal relationship between quartiles of fatigue and subsequent (1) CVD-related mortality and (2) cancer-related mortality.

Last, models were re-run with the exclusion of all participants who died within 2 years of completing SF36-VT in order to investigate the potential for reverse causality, that is, the possibility of a significant subclinical disease causing fatigue.

Analyses were undertaken using SPSS version 22.0 (IBM, Chicago, IL, US). The proportional hazard assumption was checked by introducing an interaction term of time and vitality and was found not to be violated. All reported *p*-values are for two-sided significance tests and effect sizes are expressed as hazard ratios (HR) with 95 % confidence intervals (CI).

## Results

Of the 25,639 participants who attended the baseline health assessment, complete SF36-VT data were available for 18,101. In this sample, there were a total of 4397 deaths (CVD: 1410, cancer: 1573, other causes: 1414) with over 300,322 person years of follow-up (mean 16.6 years).

Sample characteristics by SF36-VT quartile, where quartile 1 (SF36-VT: 76–100) represents low levels of fatigue and quartile 4 (SF36 VT: 0–49) represents high levels of fatigue, are described in Table 1. Quartile 1 participants were statistically more likely to be older, of female sex, married, obese, physically inactive, depressed, report higher pain, and consume more fruit and vegetables when compared to those within quartile 4. In contrast, laboratory measures such as TSH, CRP and Hb were not statistically different across fatigue quartiles and so were not considered to be confounders. All other covariates fulfilled the criteria for confounding (Additional file 1: Table S1).

**Table 1:** Sample characteristics by SF36-VT quartiles of 18,101 EPIC-Norfolk participants

Characteristic	*N*	Total	Q1 (76–100)	Q2 (65–75)	Q3 (50–64)	Q4 (0–49)	*p*-value
Age^a^ (years)	18,101	59.0 ± 9.1	59.2 ± 8.7	59.0 ± 9.1	59.2 ± 9.3	58.6 ± 9.4	0.008
Sex	18,101						<0.001
Male		7943 (43.9)	2186 (49.7)	2322 (46.0)	1842 (40.9)	1593 (38.3)	
Female		10,158 (56.1)	2209 (50.3)	2725 (54.0)	2659 (59.1)	2565 (61.7)	
Body mass index (kg/m^2^)	18,064						<0.001
<18.5		83 (0.5)	13 (0.3)	19 (0.4)	23 (0.5)	28 (0.7)	
18.5–25		7234 (40.1)	1891 (43.1)	2078 (41.2)	1721 (38.3)	1544 (37.3)	
25–30		8123 (45.0)	2029 (46.3)	2289 (45.4)	2021 (44.9)	1784 (43.1)	
>30		2624 (14.5)	451 (10.3)	654 (13.0)	732 (16.3)	787 (19.0)	
Education	18,094						<0.001
Lower than A-level		8255 (45.6)	1912 (43.5)	2232 (44.3)	2065 (45.9)	2046 (49.2)	
At least A-level		9839 (54.4)	2481 (56.5)	2812 (55.8)	2435 (54.1)	2111 (50.8)	
Married	18,009						<0.001
Yes		14,681 (81.5)	3613 (82.6)	4184 (83.4)	3648 (81.5)	3236 (78.2)	
No^b^		3328 (18.5)	763 (17.4)	834 (16.6)	830 (18.5)	901 (21.8)	
Social Class	17,757						<0.001
Manual		6725 (37.9)	1561 (36.2)	1778 (35.9)	1696 (38.4)	1690 (41.5)	
Non-manual		11,032 (62.1)	2753 (63.8)	3178 (64.1)	2716 (61.6)	2385 (58.5)	
Physical activity	18,101						<0.001
Inactive		5226 (18.7)	1019 (23.2)	1321 (26.2)	1372 (30.5)	1514 (36.4)	
Moderately inactive		5287 (23.3)	1246 (28.4)	1519 (30.1)	1324 (29.4)	1198 (28.8)	
Moderately active		4210 (29.2)	1092 (24.8)	1230 (24.4)	1022 (22.7)	886 (20.8)	
Active		3378 (28.9)	1038 (23.6)	977 (19.4)	783 (17.4)	580 (13.9)	
Depression	18,101						<0.001
Yes		2554 (14.1)	315 (7.2)	547 (10.8)	645 (14.3)	1047 (25.2)	
No		15,547 (85.9)	4080 (92.8)	4500 (89.2)	3856 (85.7)	3111 (74.8)	
Diabetes	18,101						<0.001
Yes		392 (2.2)	77 (1.8)	92 (1.8)	101 (2.2)	122 (2.9)	
No		17,709 (97.8)	4318 (98.3)	4955 (98.2)	4400 (97.8)	4036 (97.1)	
Bodily pain (SF36)	18,080	74.5 ± 23.0	87.7 ± 15.5	79.7 ± 18.8	70.7 ± 21.8	57.7 ± 24.6	<0.001
Fruit and vegetable consumption (g/day)	17,621	522.6 ± 257.0	539.8 ± 270.6	527.8 ± 245.2	517.8 ± 252.9	503.0 ± 259.3	<0.001
Alcohol consumption (g/day)	17,621	8.6 ± 12.7	9.3 ± 13.4	9.1 ± 12.8	8.2 ± 12.3	7.7 ± 12.4	<0.001
Beta blocker use	18101						<0.001
Yes		1138 (6.3)	215 (4.9)	278 (5.5)	323 (7.2)	322 (7.7)	
No		16,963 (93.7)	4180 (95.1)	4769 (94.5)	4178 (92.8)	3836 (92.3)	
Thyroid stimulating hormone (mU/L)	9761						0.265
High (≥4)		8857 (90.7)	2191 (90.35)	2445 (90.2)	2246 (91.7)	1975 (90.8)	
Low (<4)		904 (9.3)	234 (90.7)	266 (9.8)	204 (8.3)	200 (9.2)	
CRP (mg/L)	12,993	3.1 ± 6.2	3.1 ± 5.6	3.1 ± 6.4	2.9 ± 5.4	3.3 ± 7.3	0.223
Haemoglobin (g/dL)	12,971						0.886
High (≥11)		12,793 (98.6)	3138 (98.7)	3553 (98.5)	3220 (98.7)	2882 (98.6)	
Low (<11)		178 (1.37)	41 (1.3)	54 (1.5)	43 (1.3)	40 (1.4)	

p values indicate levels of significance across all quartiles

Data presented as mean (SD) for all continuous variables and number (%) for categorical variables

^a^at SF36 completion ^b^includes single, widowed, seperated and divorced

Table 2 shows the absolute risks and hazard ratios for all-cause mortality by quartiles of SF36-VT. All models consistently indicated that participants reporting the highest fatigue (SF36-VT quartile 4) were at significantly higher risk of mortality over the follow-up period than those reporting the lowest fatigue (SF36-VT quartile 1). The magnitude of this risk following exclusion of those with a history of CVD (*n* = 1533) and cancer (*n* = 1410) and then adjustment of other confounders (model C: HR 1.40, 95 % CI 1.25–1.56) was attenuated by thyroid function (model E: HR 1.26, 95 % CI 1.10–1.45) and less so by CRP (model F: HR 1.39, 95 % CI 1.21–1.60).

**Table 2:** The risks of all-cause mortality by SF36 vitality score in the EPIC-Norfolk study

Mortality	*N* (%)	SF36 vitality score quartile				*p*-value^a^
		76–100	65–75	50–64	0–49	
Model A: unadjusted full dataset HR (95 % CI)	4397/18,101 (24.3 %)	928/4395 (21.1 %)	1103/5047 (21.9 %)	1154/4501 (25.6 %)	1212/4158 (29.1 %)	<0.001
		1 (reference)	1.04 (0.96–1.14)	1.27 (1.17–1.39)	1.49 (1.36–1.62)	
Model B: unadjusted with removal of baseline CVD/cancer HR (95 % CI)	3637/16,409 (22.2 %)	825/4097 (20.1 %)	945/4657 (20.3 %)	944/4049 (23.3 %)	923/3606 (25.6 %)	<0.001
		1 (reference)	1.01 (0.92–1.11)	1.20 (1.10–1.32)	1.34 (1.22–1.47)	
Model C: confounders HR (95 % CI)	3388/15,577 (21.8 %)	771/3899 (19.8 %)	886/4442 (19.9 %)	880/3837 (22.9 %)	851/3399 (25.0 %)	<0.001
		1 (reference)	1.02 (0.93–1.13)	1.21 (1.10–1.34)	1.40 (1.25–1.56)	
Model D: confounders + Hb HR (95 % CI)	3388/15,577 (21.8 %)	771/3899 (19.8 %)	886/4442 (19.9 %)	880/3837 (22.9 %)	851/3399 (25.0 %)	<0.001
		1(reference)	1.01 (0.91–1.13)	1.24 (1.12–1.39)	1.40 (1.24–1.58)	
Model E: confounders + TSH HR (95 % CI)	3388/15,577 (21.8 %)	771/3899 (19.8 %)	886/4442 (19.9 %)	880/3837 (22.9 %)	851/3399 (25.0 %)	<0.001
		1 (reference)	0.97 (0.86–1.09)	1.23 (1.09–1.39)	1.26 (1.10–1.45)	
Model F: confounders + CRP HR (95 % CI)	3388/15,577 (21.8 %)	771/3899 (19.8 %)	886/4442 (19.9 %)	880/3837 (22.9 %)	851/3399 (25.0 %)	<0.001
		1 (reference)	1.06 (0.94–1.19)	1.24 (1.10–1.40)	1.39 (1.21–1.60)	
Model G: confounders + Hb, CRP, TSH HR (95 % CI)	3388/15,577 (21.8 %)	771/3899 (19.8 %)	886/4442 (19.9 %)	880/3837 (22.9 %)	851/3399 (25.0 %)	0.001
		1 (reference)	1.00 (0.87–1.15)	1.26 (1.09–1.46)	1.24 (1.05–1.46)	

^a^Across quartiles

Model A: unadjusted; Model B: unadjusted with removal of baseline CVD/cancer; Model C: Model B + statistically proven confounders – age, gender, body mass index, marital status, smoking, education, alcohol, social class, diet, physical activity, beta blocker, depression, pain, diabetes; Model D: Model C plus Hb adjusted; Model E: Model C plus TSH adjusted; Model F: Model C plus CRP adjusted; Model G: Model C plus Hb, CRP and TSH adjusted

*CI* confidence interval, *CRP* C-reactive protein, *CVD* cardiovascular disease, *Hb* haemoglobin, *HR* hazard ratio, *TSH* thyroid-stimulting hormone

On further inspection of disease-specific causes of mortality, participants with high fatigue were at greater risk of CVD-related death (Table 3) (model C: confounder adjusted HR 1.45, 95 % CI 1.18–1.78).

**Table 3:** The risks of cardiovascular disease related mortality by SF-36 vitality score in the EPIC-Norfolk study

Mortality	SF-36 vitality score quartiles				*p*-value^a^
	76–100	65–75	50–64	0–49	
Model A: unadjusted full dataset HR (95 % CI)	1 (reference)	0.98 (0.84–1.15)	1.25 (1.07–1.46)	1.63 (1.40–1.89)	<0.001
Model B: unadjusted with removal of baseline CVD/cancer HR (95 % CI)	1 (reference)	0.92 (0.77–1.09)	1.18 (1.00–1.40)	1.41 (1.19–1.67)	<0.001
Model C: confounders HR (95 % CI)	1 (reference)	0.93 (0.78–1.12)	1.21 (1.00–1.45)	1.45 (1.18–1.78)	<0.001
Model D: confounders + Hb HR (95 % CI)	1(reference)	0.93 (0.77–1.13)	1.21 (1.00–1.48)	1.43 (1. 15–1.78)	<0.001
Model E: confounders + TSH HR (95 % CI)	1 (reference)	0.85 (0.68–1.05)	1.22 (0.98–1.52)	1.31 (1.03–1.68)	0.001
Model F: confounders + CRP HR (95 % CI)	1 (reference)	0.96 (0.77–1.20)	1.16 (0.92–1.45)	1.38 (1.07–1.79)	0.021
Model G: confounders + Hb, CRP, TSH HR (95 % CI)	1 (reference)	0.84 (0.64–1.09)	1.20 (0.92–1.56)	1.26 (0.93–1.70)	0.025

^a^Across quartiles

Model A: unadjusted; Model B: unadjusted with removal of baseline CVD/cancer; Model C: Model B + statistically proven confounders – age, gender, body mass index, marital status, smoking, education, alcohol, social class, diet, physical activity, beta blocker, depression, pain, diabetes; Model D: Model C plus Hb adjusted; Model E: Model C plus TSH adjusted; Model F: Model C plus CRP adjusted; Model G: Model C plus Hb, CRP and TSH adjusted

*CI* confidence interval, *CRP* C-reactive protein, *CVD* cardiovascular disease, *Hb* haemoglobin, *HR* hazard ratio, *TSH* thyroid-stimulting hormone

In contrast, no significantly increased risk of cancer-related deaths was observed in any of the adjusted models (Table 4). Because cancer and CVD accounted for the majority of deaths, the apparent excess of mortality associated with fatigue appears to be mostly driven by CVD-related deaths. As with all-cause mortality, thyroid function (model E: HR 1.31, 95 % CI 1.03–1.68) and CRP (model F: HR 1.38, 95 % CI 1.07–1.79) attenuated the association between high fatigue and mortality over the period of follow-up. Despite adjusting for all putative confounders and mechanisms, the risk remained pronounced for both all-cause mortality (fully adjusted: HR 1.24, 95 % CI 1.05–1.46) and a similar magnitude of risk for CVD-related mortality (fully adjusted: HR 1.26, 95 % 0.93–1.70).

**Table 4:** The risks of cancer related mortality by SF36 vitality score in the EPIC-Norfolk study

Mortality	SF36 vitality score quartiles				*p* value^a^
	76–100	65–75	50–64	0–49	
Model A: unadjusted full dataset HR (95 % CI)	1 (reference)	0.97 (0.84–1.16)	1.16 (1.01–1.33)	1.10 (0.95–1.27)	0.043
Model B: unadjusted with removal of baseline CVD/cancer HR (95 % CI)	1 (reference)	0.95 (0.82–1.10)	1.10 (0.95–1.28)	0.98 (0.83–1.15)	0.237
Model C: confounders HR (95 % CI)	1 (reference)	0.96 (0.83–1.13)	1.14 (0.96–1.34)	1.09 (0.90–1.32)	0.185
Model D: confounders + Hb HR (95 % CI)	1(reference)	0.94 (0.79–1.11)	1.16 (0.97–1.38)	1.12 (0.91–1.38)	0.086
Model E: confounders + TSH HR (95 % CI)	1 (reference)	0.91 (0.75–1.10)	1.13 (0.93–1.38)	0.98 (0.77–1.24)	0.132
Model F: confounders + CRP HR (95 % CI)	1 (reference)	1.06 (0.88–1.27)	1.18 (0.97–1.44)	1.09 (0.87–1.37)	0.385
Model G: confounders + Hb, CRP,TSH HR (95 % CI)	1 (reference)	1.04 (0.84–1.29)	1.19 (0.95–1.51)	0.86 (0.65–1.15)	0.081

^a^Across quartiles

Model A: unadjusted; Model B: unadjusted with removal of baseline CVD/cancer; Model C: Model B + statistically proven confounders – age, gender, body mass index, marital status, smoking, education, alcohol, social class, diet, physical activity, beta blocker, depression, pain, diabetes; Model D: Model C plus Hb adjusted; Model E: Model C plus TSH adjusted; Model F: Model C plus CRP adjusted; Model G: Model C plus Hb, CRP and TSH adjusted

*CI* confidence interval, *CRP* C-reactive protein, *CVD* cardiovascular disease, *Hb* haemoglobin, *HR* hazard ratio, *TSH* thyroid-stimulting hormone

Finally, the effect of excluding all deaths occurring within 2 years of follow-up (*n* = 4) upon the final models was negligible.

## Discussion

In this large population-based prospective study with long-term follow-up, fatigue was significantly associated with premature mortality, even after adjusting for multiple potential confounders. Compared with those in the lowest quartile of reported fatigue levels, participants in the highest quarter of reported fatigue levels had a 40 % higher risk of premature mortality. This association was observed for CVD-related, but not cancer-related, deaths. Thyroid status and CRP level attenuated these associations although these do not entirely explain the mechanisms which underpin this concerning observation.

As with all studies of this nature, the results must be considered in the context of some possible limitations.

First, although general practices in the UK are considered ideal population sample frames (because almost all people are registered to a general practitioner through the National Health Service), only 40–45 % of registrants provided baseline data, of which approximately 30 % did not complete the SF36-VT questionnaire. Nonetheless, previous descriptions of this cohort have failed to identify significant differences in population characteristics when compared to other national samples, except for a slightly lower prevalence of smokers [12], and no material differences in background characteristics were identified between SF36-VT responders and non-responders. Overall, these results appear generalisable when applied to middle- to old-aged members of the general population, although, owing to the study design, they are unlikely to fully apply to typical healthcare attendees principally presenting with significant fatigue. It is anticipated that such persons will complain of more severe levels of fatigue compared to this study’s modest definition of high fatigue (a self-reported score within the top 25 % of the general population over a period of 1 month). Chronic fatigue disorders, for example, are frequently characterised as disabling and lasting for >6 months [21]. Because our data evidences a consistent dose response between fatigue severity and mortality, these patients are likely to be at even greater risk of premature death in comparison to the population described in this study. These data may, however, not be used to infer the prognosis of patients with chronic fatigue syndrome because information on total fatigue duration was not measured and this may also represent a distinct disorder. Indeed, recent evidence suggests that such patients have a different risk factor profile compared to the general population in terms of mortality causes [22].

Second, there is the potential for pre-existing baseline morbidities, especially CVD, to exaggerate the true association between fatigue and premature mortality. We attempted to address such reverse causality within the analysis by excluding participants who reported baseline CVD and cancer. To further exclude the influence of possible subclinical disease, deaths within 2 years were excluded and baseline aspirin use, a surrogate marker of high CVD risk, was submitted as a covariate. Neither resulted in a meaningful modification of the final models.

Third, there are potential measurement errors in the assessment of exposures. We only used a single measure at one time point to assess each confounder and so did not take into account possible changes over the follow-up period. That said, such random measurement error would probably attenuate any associations observed, so the estimated differences in risk are likely to be larger than those observed.

In relation to other prognostic factors of mortality, which are already considered key targets for intervention in modern-day health services, the observed unadjusted mortality risk of high fatigue (HR 1.63, 95 % CI 1.40–1.89) is similar to being a current smoker (HR 1.35, 95 % CI 1.24–1.48, see Additional file 1: Table S2) in this same sample (although direct comparisons of HRs are not possible owing to differences in the measurement scale). These results are even more meaningful from a public health perspective given the highly prevalent nature of fatigue. Hardy et al. [10] and Moreh et al. [9] also reported similarly sized effects of fatigue on mortality (adjusted HR 1.40, 95 % CI 1.08–1.93 and 1.31, 0.92–1.86, respectively) although their study populations were restricted to the elderly and were small (*n* = 492 and *n* = 605, respectively), so did not determine cause-specific mortality [9, 10]. Furthermore, both utilised an unvalidated single item question to measure fatigue, failed to investigate key putative mechanisms such as thyroid status and inflammation, and did not account for the possibility of reverse causality as we have.

Knowledge regarding the mechanisms which cause fatigue is limited and the precise pathophysiology of fatigue has yet to be elucidated. Possible insights may be derived from research examining the concept of ‘burnout’ – a multidimensional construct consisting of emotional exhaustion, cognitive weariness and physical fatigue. Intriguingly, this is also associated with all-cause mortality, in particular CVD [23]. This state has been characterised by reduced hypothalamic pituitary adrenal axis responses [24], low grade inflammation [25] and impaired fibrinolytic capacities [26], all of which have also been independently associated with CVD [27–29]. It is interesting that our study also identified inflammation, as measured by CRP, to be a potentially relevant mechanism. We have also identified the importance of TSH. Of the multiple functions of this hormone, its positive influence on the basal metabolic rate and on catecholamine sensitivity best explain why low levels commonly manifest as fatigue [30]. Furthermore, the hypothyroid state is associated with accelerated atherosclerosis, systemic vascular resistance and subsequent CVD [31]. Though this study does not directly inform whether thyroxine replacement (in those with high abnormal TSH level) would attenuate the mortality risk of fatigue, the data in combination with existing biological knowledge does strongly allude to this.

The persisting significant risk despite adjustment of almost all routinely considered associations highlights the need for greater therapeutic options in this field. This cannot be achieved without further research in this area to unravel the likely complex mechanisms. Based on our findings, such biological mechanisms may be shared with CVD, providing a starting point for further investigation, but other behavioural and psychosocial factors, for example work-shift patterns, should also be considered.

Being in the highest quartile for self-reported fatigue is significantly predictive of death and on a par with recognised risk factors such as active smoking. The risk is likely to be even greater for those presenting with more severe symptoms and is especially associated with CVD-related death. This study therefore indicates the potential seriousness of this frequently dismissed symptom [7]. The attenuation of risk by both behavioural and clinical factors in our models suggests a potential benefit of a comprehensive bio-psychosocial approach. In the future, fatigued persons may benefit from both non-pharmacological interventions, for example physical activity, and pharmacological interventions, for example anti-inflammatory agents.

## Conclusions

This study presents the first reliable evidence linking the common symptom of fatigue with excess mortality. Patients have long recognised the importance of fatigue [7]. The biomedical community should now align with this patient priority and more optimally research this problem because further elucidation and targeting of the mechanisms may not only improve crucial patient reported outcomes but conceivably improve life longevity as well.

## Abbreviations

CI, confidence interval; CRP, C-reactive protein; CVD, cardiovascular disease; EPIC, European Prospective Investigation into Cancer and Nutrition; Hb, haemoglobin; HR, hazard ratio; ICD, International Classification of Disease; ONS, Office for National Statistics; SF36-VT, Short form 36 vitality, TSH, thyroid-stimulating hormone

## Additional file

Additional file 1: Table S1. Associations between baseline covariates and mortality. **Table S2.** The risks of all-cause mortality by smoking. (DOCX 33 kb)
