# Supplementary material for: Fatigue is associated with excess mortality in the general population: results from the EPIC-Norfolk study
Source: BMC Med. 2016 Aug 20;14:122. doi: 10.1186/s12916-016-0662-y (PMC4992307; doi:10.1186/s12916-016-0662-y)
Supplement: Additional file 1: Table S1. — Associations between baseline covariates and mortality. Table S2. The risks of all-cause mortality by smoking. (DOCX 33 kb) [file 12916_2016_662_MOESM1_ESM.docx]

Additional file 1

**Table S1** Associations between baseline covariates and mortality

| Characteristic | N | Total | alive | dead | P-value |
| --- | --- | --- | --- | --- | --- |
| Age (per 5 years) | 18101 |  |  |  | **<0.001^#^** |
| 40-44 |  | 709 | 696(5.1) | 13(0.3) |  |
| 45-49 |  | 3003 | 2863(20.9) | 140(3.2) |  |
| 50-54 |  | 2925 | 2674(19.5) | 251(5.7) |  |
| 55-59 |  | 2896 | 2533(18.5) | 363(8.3) |  |
| 60-64 |  | 2943 | 2237(16.3) | 706(16.1) |  |
| 65-69 |  | 2830 | 1709(12.5) | 1121(25.5) |  |
| 70-74 |  | 2287 | 867(6.3) | 1420(32.3) |  |
| 75-80 |  | 508 | 125(0.9) | 383(8.7) |  |
| Sex | 18101 |  |  |  | **<0.001^#^** |
| Male |  | 7943 | 5536(40.4) | 2407(54.7) |  |
| Female |  | 10158 | 8168(59.6) | 1990(45.3) |  |
| BMI (kg/m^2^) | 18064 |  |  |  | **<0.001^#^** |
| <18.5 |  | 83 | 66(0.5) | 17(0.4) |  |
| 18.5-25 |  | 7234 | 5702(41.7) | 1532(34.9) |  |
| 25-30 |  | 8123 | 6069(44.4) | 2054(46.8) |  |
| >30 |  | 2624 | 1842(13.5) | 782(17.8) |  |
| Education | 18094 |  |  |  | **<0.001^#^** |
| Lower than A-level |  | 8255 | 5954(43.5) | 2301(52.3) |  |
| At least A-level |  | 9839 | 7744(56.5) | 2095(47.7) |  |
| Married | 18009 |  |  |  | **<0.001^#^** |
| Yes |  | 14681 | 11362(83.4) | 3319(75.8) |  |
| No^b^ |  | 3328 | 2267(16.6) | 1061(24.2) |  |
| Smoking | 18101 |  |  |  | **<0.001^#^** |
| Not current smoker |  | 16236 | 12402(90.5) | 3834(87.2) |  |
| Current smoker |  | 1865 | 1302(9.5) | 563(12.8) |  |
| Social Class | 17757 |  |  |  | **0.006^#^** |
| Manual |  | 6725 | 5037(37.3) | 1688(39.7) |  |
| Non-manual |  | 11032 | 8464(62.7) | 2568(60.3) |  |
| Physical activity | 18101 |  |  |  | **<0.001^#^** |
| Inactive |  | 5226 | 3374(24.6) | 1852(42.1) |  |
| Moderately inactive |  | 5287 | 4111(30.0) | 1176(26.7) |  |
| Moderately active |  | 4210 | 3422(25.0) | 788(17.9) |  |
| Active |  | 3378 | 2797(20.4) | 581(13.2) |  |
| Depression | 18101 |  |  |  | **<0.001^#^** |
| No |  | 15547 | 11671(85.2) | 3876(88.2) |  |
| Yes |  | 2554 | 2033(14.8) | 521(11.8) |  |
| Diabetes | 18101 |  |  |  | **<0.001^#^** |
| No |  | 17709 | 13518(98.6) | 4191(95.3) |  |
| Yes |  | 392 | 186(1.4) | 206(4.7) |  |
| Bodily pain (SF-36) | 18080 | 74.09±23.29 | 75.30±22.64 | 70.33±24.86 | **<0.001^*^** |
| Physical function (SF-36) | 17882 | 78.70±22.43 | 82.13±19.88 | 67.89±26.26 | **<0.001^*^** |
| Fruit and vegetable consumption (g/day) | 17621 | 522.55±257.01 | 525.77±258.04 | 512.46±253.54 | **0.003^*^** |
| Alcohol consumption (g/day) | 17621 | 8.60±12.75 | 8.60±12.47 | 8.58±13.57 | **<0.001^$^** |
| Beta blocker use | 18101 |  |  |  | **<0.001** |
| No |  | 16963 | 13001(94.9) | 3962(90.1) |  |
| Yes |  | 1138 | 703(5.1) | 435(9.9) |  |
| Aspirin use | 18101 |  |  |  | **<0.001^#^** |
| No |  | 16745 | 12945(94.5) | 3800(86.4) |  |
| Yes |  | 1356 | 759(5.5) | 597(13.6) |  |
| Thyroid stimulating hormone (mU/L) | 9761 | 2.39±4.11 | 2.38±4.11 | 2.42±4.52 | **0.767^#^** |
| CRP (mg/L) | 12993 | 3.10±6.20 | 3.11±6.22 | 3.06±6.12 | **0.689^*^** |
| Haemoglobin (g/dL) | 12971 | 13.85±1.31 | 13.87±1.31 | 13.81±1.33 | **0.011^*^** |

**# Chi-squared test *ANOVA $ Kruskal-Wallis test**

**Associations between baseline covariates and SF36 vitality**

| Characteristic | | N | Total | Q1 (76-100) | Q2 (65-75) | Q3 (50-64) | Q4 (0-49) | P-value |
| --- | --- | --- | --- | --- | --- | --- | --- | --- |
| Age (per 5 years) | | 18101 |  |  |  |  |  | **<0.001^#^** |
| 40-44 |  | 709 | 141(3.2) | 208(4.1) | 184(4.1) | 176(4.2) |  |  |
| 45-49 |  | 3003 | 671(15.3) | 826(16.4) | 716(15.9) | 790(19.0) |  |  |
| 50-54 |  | 2925 | 692(15.7) | 797(15.8) | 739(16.4) | 697(16.8) |  |  |
| 55-59 |  | 2896 | 766(17.4) | 827(16.4) | 676(15.0) | 627(15.1) |  |  |
| 60-64 |  | 2943 | 812(18.5) | 847(16.8) | 716(15.9) | 568(13.7) |  |  |
| 65-69 |  | 2830 | 714(16.2) | 805(16.0) | 732(16.3) | 579(13.9) |  |  |
| 70-74 |  | 2287 | 506(11.5) | 608(12.0) | 600(13.2) | 573(13.8) |  |  |
| 75-80 |  | 508 | 93(2.1) | 129(2.6) | 138(3.1) | 148(3.6) |  |  |
| Sex | | 18101 |  |  |  |  |  | **<0.001^#^** |
| Male | |  | 7943(43.9) | 2186(49.7) | 2322(46.0) | 1842(40.9) | 1593(38.3) |  |
| Female | |  | 10158(56.1) | 2209(50.3) | 2725(54.0) | 2659(59.1) | 2565(61.7) |  |
| BMI (kg/m^2^) | | 18064 |  |  |  |  |  | **<0.001^#^** |
| <18.5 | |  | 83(0.5) | 13(0.3) | 19(0.4) | 23(0.5) | 28(0.7) |  |
| 18.5-25 | |  | 7234(40.1) | 1891(43.1) | 2078(41.2) | 1721(38.3) | 1544(37.3) |  |
| 25-30 | |  | 8123(45.0) | 2029(46.3) | 2289(45.4) | 2021(44.9) | 1784(43.1) |  |
| >30 | |  | 2624(14.5) | 451(10.3) | 654(13.0) | 732(16.3) | 787(19.0) |  |
| Education | | 18094 |  |  |  |  |  | **<0.001^#^** |
| Lower than A-level | |  | 8255(45.6) | 1912(43.5) | 2232(44.3) | 2065(45.9) | 2046(49.2) |  |
| At least A-level | |  | 9839(54.4) | 2481(56.5) | 2812(55.8) | 2435(54.1) | 2111(50.8) |  |
| Married | | 18009 |  |  |  |  |  | **<0.001^#^** |
| Yes | |  | 14681(81.5) | 3613(82.6) | 4184(83.4) | 3648(81.5) | 3236(78.2) |  |
| No^b^ | |  | 3328(18.5) | 763(17.4) | 834(16.6) | 830(18.5) | 901(21.8) |  |
| Smoking | | 18101 |  |  |  |  |  | **<0.001^#^** |
| Not current smoker | |  | 16236 | 3987(90.7) | 4600(91.1) | 4051(90.0) | 3598(86.5) |  |
| Current smoker | |  | 1865 | 408(9.3) | 447(8.9) | 450(10.0) | 560(13.5) |  |
| Social Class | | 17757 |  |  |  |  |  | **<0.001^#^** |
| Manual | |  | 6725(37.9) | 1561(36.2) | 1778(35.9) | 1696(38.4) | 1690(41.5) |  |
| Non-manual | |  | 11032(62.1) | 2753(63.8) | 3178(64.1) | 2716(61.6) | 2385(58.5) |  |
| Physical activity | | 18101 |  |  |  |  |  | **<0.001^#^** |
| Inactive | |  | 5226(18.7) | 1019(23.2) | 1321(26.2) | 1372(30.5) | 1514(36.4) |  |
| Moderately inactive | |  | 5287(23.3) | 1246(28.4) | 1519(30.1) | 1324(29.4) | 1198(28.8) |  |
| Moderately active | |  | 4210(29.2) | 1092(24.8) | 1230(24.4) | 1022(22.7) | 886(20.8) |  |
| Active | |  | 3378(28.9) | 1038(23.6) | 977(19.4) | 783(17.4) | 580(13.9) |  |
| Depression | | 18101 |  |  |  |  |  | **<0.001^#^** |
| Yes | |  | 2554(14.1) | 315(7.2) | 547(10.8) | 645(14.3) | 1047(25.2) |  |
| No | |  | 15547(85.9) | 4080(92.8) | 4500(89.2) | 3856(85.7) | 3111(74.8) |  |
| Diabetes | | 18101 |  |  |  |  |  | **<0.001^#^** |
| Yes | |  | 392(2.2) | 77(1.8) | 92(1.8) | 101(2.2) | 122(2.9) |  |
| No | |  | 17709(97.8) | 4318(98.3) | 4955(98.2) | 4400(97.8) | 4036(97.1) |  |
| Bodily pain (SF-36) | | 18080 | 74.5±23.0 | 87.7±15.5 | 79.7±18.8 | 70.7±21.8 | 57.7±24.6 | **<0.001^*^** |
| Physical function ( SF-36) | | 17882 | 78.7±22.4 | 89.7±13.7 | 84.5±16.5 | 76.1±21.1 | 62.8±27.4 | **<0.001^*^** |
| Fruit and vegetable consumption (g/day) | | 17621 | 522.6±257.0 | 539.8±270.6 | 527.8±245.2 | 517.8±252.9 | 503.0±259.3 | **<0.001^*^** |
| Alcohol consumption (g/day) | | 17621 | 8.6±12.7 | 9.3±13.4 | 9.1±12.8 | 8.2±12.3 | 7.7±12.4 | **<0.001^$^** |
| Beta blocker use | | 18101 |  |  |  |  |  | **<0.001^#^** |
| Yes | |  | 1138(6.3) | 215(4.9) | 278(5.5) | 323(7.2) | 322(7.7) |  |
| No | |  | 16963(93.7) | 4180(95.1) | 4769(94.5) | 4178(92.8) | 3836(92.3) |  |
| Aspirin use | | 18101 |  |  |  |  |  | **<0.001^#^** |
| Yes | |  | 1356 | 248(5.6) | 363(7.2) | 381(8.5) | 364(8.8) |  |
| No | |  | 16745 | 4147(94.4) | 4684(92.8) | 4120(91.5) | 3794(91.2) |  |
| Thyroid stimulating hormone (mU/L) | | 9761 | 2.4±4.7 | 2.4±3.7 | 2.44.5 | 2.3±4.0 | 2.4±4.7 | **0.401^$^** |
| CRP (mg/L) | | 12993 | 3.1±6.2 | 3.1±5.6 | 3.1±6.4 | 2.9±5.4 | 3.3±7.3 | **0.223^*^** |
| Haemoglobin (g/dL) | | 12971 | 13.9±1.3 | 13.9±1.3 | 13.8±1.3 | 13.8±1.3 | 13.9±1.3 | **0.765^*^** |

**Table S2: The risks of all-cause mortality by smoking**

**# Chi-squared test *ANOVA $ Kruskal-Wallis test**

| Mortality | Non-current smoker | Current smoker | p-value |
| --- | --- | --- | --- |
| All-cause, n | 16236 | 1865 |  |
| Rate/100 (events) | 23.61 (3834) | 30.19 (563) | <0.001 |
| Unadjusted HR | 1(reference) | 1.35(1.24-1.48) | <0.001 |
